# Association of bone turnover C-terminal telopeptide levels, 25-hydroxyvitamin D concentrations and calcium intake in Malaysian adolescents

**DOI:** 10.3389/fnut.2025.1666496

**Published:** 2025-10-28

**Authors:** Hazreen Abdul Majid, Muzaitul Akma Mustapa Kamal Basha, Marjolijn C. E. Bragt, Ellen G. H. M. van den Heuve, Muhammad Yazid Jalaludin

**Affiliations:** ^1^Centre for Preventive Health Care, Health Sciences University, Bournemouth, United Kingdom; ^2^International Islamic University Malaysia Kulliyyah of Medicine, Kuantan, Malaysia; ^3^FrieslandCampina, Stationsplein, Amersfoort, Netherlands; ^4^Department of Paediatrics, Faculty of Medicine, University of Malaya, Kuala Lumpur, Malaysia

**Keywords:** bone turnover, C-terminal telopeptide (CTX), Vitamin D, calcium intake, adolescents

## Abstract

**Introduction:**

The C-terminal telopeptide (CTX), vitamin D, and calcium intake are key factors in bone health research. This study aimed to investigate these associations among adolescents.

**Methods:**

A cross-sectional study was conducted involving 1,234 students (15-year-olds) from public schools in Selangor, Perak, and Kuala Lumpur, Malaysia. The levels of CTX, 25-hydroxyvitamin D (25(OH)D), and several biomarkers were measured. The seven-day diet history was applied to the habitual dietary intake of the participants.

**Results:**

A generalized linear model was used to quantify the relationship between 25(OH)D concentrations and calcium intake with CTX levels. The results showed that the CTX log concentration was higher in females than males (mean±SD; 1.32±0.47 ng/mL vs. 1.24±0.44 ng/mL). Regarding vitamin D, the percentage of participants with 25(OH)D concentrations below 50 nmol/L was higher among females than males (91.9% vs. 45.4%). In terms of calcium intake, all participants consumed less than 50% (indicating low calcium intake) of the recommended amount, with intakes ranging from 282.0 mg/day to 543.1 mg/day. No significant association was found between 25(OH)D levels below 50 nmol/L and CTX concentrations (*p*-value > 0.1), but there was a significant association between CTX levels and calcium intake status (β=0.161, *p*-value < 0.01).

**Conclusion:**

This study suggests that higher bone turnover, indicated by elevated CTX levels, is associated with low calcium intake but not with low 25(OH)D levels.

## 1 Introduction

Bone is a dynamic organ that serves mechanical and homeostatic functions. It undergoes a continual self-regeneration process called remodeling. Remodeling removes old bone and replaces it with new bone. This regenerative process occurs in distinct areas of bone known as bone metabolic units (BMU). Within each BMU, bone formation by osteoblasts and bone resorption by osteoclasts are coupled tightly and in a delicate balance to maintain bone mass and strength. Adolescence is a critical period for peak bone mass accrual (1). It is well-documented that male adolescents were seen to have achieved a higher peak bone mass than females due to differences in body size, muscle mass, and puberty (2). Evidence has shown that any interference with bone mass metabolism (either bone formation or resorption) during adolescence has been linked to the risks of fracture and increases the risk of fracture and skeletal disease later in life (3–5).

Serum cross-linked C-telopeptide of type I collagen (CTX) is one of the bone biochemical markers of bone turnover, a biomarker of osteoclast activity whereby it is used to assess bone resorption (6). In regard to biochemical markers of bone turnover, it can be presented in two: (i) markers of bone formation, which reflect osteoblast activity and are byproducts of collagen synthesis, matrix proteins or osteoblastic enzymes, and (ii) markers of bone resorption, which reflect osteoclast activity and are for the most part degradation products of type I collagen (6, 7). Vitamin D has been demonstrated to play an essential role in bone health both indirectly and directly (8–10). Indirectly, the function of vitamin D in bone is to maintain calcium homeostasis by increasing intestinal absorption of calcium (9, 10) and directly via the vitamin D receptor system (8).

Vitamin D is essential for optimal bone health, as it plays a crucial role in regulating calcium and phosphorus metabolism, which are vital for bone formation and maintenance. Adequate levels of vitamin D facilitate the absorption of calcium from the intestine, ensuring that the body has sufficient calcium to support bone mineralization. Furthermore, vitamin D directly influences osteoblast function, promoting bone formation while also inhibiting excessive bone resorption by osteoclasts. This balance is critical, especially during adolescence, a key period for peak bone mass development. Insufficient vitamin D levels can lead to decreased bone density, increasing the risk of fractures and long-term skeletal disorders. Thus, maintaining adequate vitamin D status is fundamental for the development of strong, healthy bones during the crucial growth phases of adolescence.

Even though there is substantial data on biochemical markers of bone turnover, there is limited evidence of the C-terminal telopeptide (CTX) of bone turnover in adolescent populations with low calcium and vitamin D concentrations. The importance of vitamin D together with dietary calcium intake for bone health has been confirmed by several meta-analyses (9, 10). It is known that the primary role of calcium is to help build and maintain the bones, whereas vitamin D functions to regulate intestinal calcium absorption by stimulating bone resorption to maintain the serum calcium concentration (11).

Hence, a lack of vitamin D may affect bone growth and impair the acquisition of peak bone mass, particularly during adolescence. Furthermore, the concentration of circulating 25(OH)D required for optimal bone health in adolescents is uncertain. Several observational studies have reported a negative association between serum 25(OH)D and biomarkers of bone resorption among children and adolescents with vitamin D deficiency or insufficiency (12, 13), though some studies revealed no significant association between serum 25(OH)D and bone biomarkers (14, 15). Pludowski et al. (5) have described in the vitamin D supplement guidelines that the vitamin D concentration (25(OH)D < 50 nmol/L) has clinical implications for bone health disease. Vitamin D (25(OH)D < 50 nmol/L) influences 20 to 40% of peak bone mass, which leads to high bone resorption and defects in bone formation (4, 5, 16, 17).

Evidence from Malaysia and Southeast Asia region remains limited, despite the high prevalence of both low calcium intake and vitamin D deficiency among adolescents. Presently, most Malaysian adolescents (91.7%) fail to meet the Recommended Nutrient intake for dietary calcium, which is set at 1000 mg/day, and have insufficient levels of vitamin D (25(OH)D < 50 nmol/L) (18). Suriawati et al. (18) reported that the average calcium intake among 13-year-old Malaysians is only 370 ± 12 mg/day. Additionally, Quah et al. (20) found that 15-year-old Malaysians have serum 25(OH)D below 50 nmol/L (92.0%). Various factors, including sex, ethnicity, living area, obesity, skin type, clothes style, and vitamin D dietary intake, have been identified as influences on vitamin D concentrations among Malaysian adolescents (19, 20). Based on these findings, we hypothesized that among 15-year-old Malaysian adolescents, lower dietary calcium intake and insufficient serum concentrations of 25-hydroxyvitamin D (25(OH)D) are associated with increased levels of C-terminal telopeptide (CTX), indicating higher bone resorption and impaired bone turnover, ultimately affecting overall bone health. Therefore, this study aimed to investigate the association of bone turnover (CTX) with low calcium intake and 25(OH)D concentrations among 15-year-old Malaysian adolescents. This study is important as it examined these associations within a large, multi-ethnic Malaysian adolescent cohort, providing region-specific insights including behavior, participants characteristics and clothing attire which may explore further serum vitamin D concentrations that may influence CTX level. This will complement data from European and other Asian studies.

## 2 Materials and methods

### 2.1 Study design and sampling method

This was a cross-sectional study of 15-year-old adolescents from public schools under the Malaysian Health and Adolescents Longitudinal Research Team Cohort study (MyHeARTs). A multistage stratified cluster sampling method was applied to ensure adequate representation of urban and rural settings. The sample was multi-ethnic and drawn from diverse schools. A total of 1,234 adolescents were recruited from public secondary schools in Selangor, Perak, and the Federal Territory of Kuala Lumpur (WPKL). Boarding, religious, and vernacular schools were excluded because these schools are not representative of the type of Malaysian schools most students attend.

Participation was voluntary, and participants should be able to read and understand the national language, Malay, to participate in the study. Participants were excluded from the analysis for the following reasons: (1) refused to take part and without parental consent; (2) had a current endocrine disorder (e.g., hypoparathyroidism, hyperparathyroidism, rickets, osteoporosis, diabetes mellitus); (3) were on any prescription medications that affect bone metabolism (such as oral/parenteral steroid, anti-epileptic, anti-estrogens, anti-retroviral drugs, cytotoxic agents); (4) had a systemic medical illness (e.g., chronic kidney disease, end-stage liver disease, malabsorption disease, malignancy).

### 2.2 Ethics statement

Ethical approval was obtained from the Medical Ethics Committee, with MEC Ref. No: 896.34 and the National Medical Research Register number was 14–376-20486. Written information about the study and consent forms were given to the participants and their parents/guardians. Written informed consent was obtained from the parent or guardian, and all participating children were also required to sign an assent form. Each participant was given a unique identification number to ensure anonymity. The study protocol was published in the previous article (21).

### 2.3 Questionnaire

#### 2.3.1 Dietary assessment

Apart from sociodemographic information (such as sex, age, ethnicity, residency, and school location), the seven-day diet history was applied to the habitual dietary intake of the participants. To improve reliability of 7-day diet history interview, trained dietitians conducted interviews with audit checks, and the tool was pretested before data collection. The seven-day diet history was chosen for this study as this method has produced more valid estimates of adolescents’ energy and dietary intake compared to other methods. The tool was pretested on 40 participants from two different schools (one school each from an urban and rural area, respectively). For the actual study, seven qualified and trained dietitians used open-ended interviews with the participants to collect information on dietary intake over the previous seven-day period. The dietary assessment approach is consistent with validated protocols used in Malaysia (21) and is comparable to the validated FFQ derived from the MyHeARTs study (42). The calcium intake was calculated using the Nutritionist Pro™ Diet Analysis (Axxya Systems, Woodinville, WA, United States) software with the Nutrient Composition of Malaysian Food Database guidance for the dietary calcium intake.

#### 2.3.2 Sun exposure, protective behavior, and skin type

Sun exposure assessment was adapted from the ‘Sun Exposure and Sun Protection Practices for Behavioral and Epidemiologic Research’ (22). The participants were asked to fill out a questionnaire that included questions about their skin pigmentation as measured by the Fitzpatrick Scale (23), the amount of time they spent outside during the week and on weekends, the timing of outdoor activities, and the amount of body surface area exposed to sunlight. Further, sun-protective behavior was obtained from the participants, such as sunscreen protection, wearing a long-sleeved shirt, staying in the shade or under an umbrella, wearing a hat or veil, and wearing sunglasses. Before data collection, the questionnaire was assessed for face validity and test reliability by administering it to 50 adolescents (not included in this study).

#### 2.3.3 Anthropometric measurements

All the anthropometric measurements were done by trained personnel. The subjects were required to empty their bladder before their body measurements were taken. The weights of the subjects were measured using a digital electronic weighing scale (Seca 813, Seca, Birmingham, UK) and were recorded to the nearest decimal fraction of a kilogram (0.1 kg). The heights of the subjects without socks and shoes were measured to the nearest 0.1 cm using a calibrated vertical stadiometer (Seca Portable 217, Seca, Birmingham, United Kingdom). The body fat was analyzed using a portable body composition analyzer (Tanita SC- 240 MA Portable Body Composition Analyzer, Tanita Europe B. V., Amsterdam, The Netherlands). Body mass index (BMI) was calculated as weight in kilograms divided by the square of height in meters. BMI was further categorized based on IOTF cut-off points (overweight: 21.91 kg/m2 for males and 22.58 kg/m2 for females; obesity: 26.84 kg/m2 for males and 27.76 kg/m2 for females) (24). Body fat percentage was measured using a Tanita Portable Body Composition Analyzer SC-240 MA and expressed as a percentage (a method validated for Malaysian children by a previous study) (25). Then, the body fat percentage was categorized into the non-obese and obese groups (20).

#### 2.3.4 Biochemical measurements

Certified phlebotomists collected blood samples in the morning into plain tubes after an overnight fast of at least 10 h. The samples were temporarily stored at four degrees Celsius in a cool box immediately after the blood was taken. All the samples were centrifuged at 3500 revolutions per minute for 10 min to get the serum on the same day the blood was taken. Next, the serum was kept and stored in a − 80-degree Celsius freezer (Thermo Fisher Scientific 702, United States). Then, the serum was sent to an International Organization for Standardization (ISO) certified hospital pathology laboratory for 25(OH)D and intact parathyroid hormone (PTH) analyses. The 25(OH)D was analyzed using an ADVIA Centaur vitamin D assay, an automated direct competitive chemiluminescent immunoassay that detects 25(OH)D in plasma. This assay uses a proprietary releasing reagent and a monoclonal antibody. The lower limit of detection of the assay was 3.2 ng/mL (8.0 nmol/L). The inter-and intra-assay coefficients of variability for the lower concentration mean of 13.6 ng/mL (34 nmol/L) were 11.9 and 4.7%, respectively. The inter- and intra-assay coefficients of variability for the upper concentration mean of 114.1 ng/mL (285.2 nmol/L) were 4.2 and 3.0%, respectively. Meanwhile, the ELISA Kit for Cross-Linked C-Telopeptide of Type I Collagen (CTXI) (Cloud-Clone Corp, United States) was used to measure the CTX concentrations in plasma, which is the competitive inhibition test method. Analysis was done according to the test kit protocol. The detection range was 123.5–10,000 pg./mL (0.123 ng/mL – 10 ng/mL), with a sensitivity of less than 50.5 pg./mL (0.05 ng/mL). The quality check was conducted in the laboratory for about 10% of the sample randomly and the results were consistent (unpublished).

### 2.4 Variable definitions

#### 2.4.1 Vitamin D (25(OH)D)

To date, there has been no agreement on the reference interval for vitamin D status in adolescents (20). For this study, based on the clinical implications for skeletal health disease and non-skeletal health diseases which have been described in the Vitamin D supplement guidelines by Pludowski et al. (5), serum 25(OH)D < 50 nmol/L was defined as vitamin D deficiency. To standardize the concentration of vitamin D, the cut-off points of <37.4 nmol/L and 37.5–49.9 nmol/L also were presented for comparison between previous studies (19, 21).

#### 2.4.2 The CTX concentrations

The CTX concentration in plasma was treated as a continuous variable in unit ng/mL.

#### 2.4.3 Dietary calcium intake

In this study, the descriptive analysis showed that the calcium intake among the study population was less than 50% of the recommended nutrient intake. Thus, we presented the calcium intake concentrations by quartiles (Qs) of the calcium intake study sample (Q1 ≤ 347.3 mg/day; Q2 = 347.4–412.5 mg/day; Q3 ≥ 412.6 mg/day) with the minimum = 282.0 mg/day and maximum = 543.1 mg/day.

### 2.5 Statistical analysis

Data such as sociodemographic, anthropometric, and biochemical measurements were analyzed using the SPSS Statistics for Windows program, version 25.0 (Armonk, NY, United States). Categorical variables (sex, ethnicity, residence, body fatness) were described as frequencies and percentages. To determine the association, it was noticeable that stages 1 and 2 in the pubertal status factor had ‘small frequencies’ in each; therefore, the data were combined in inference statistics analyses. The analysis was also performed if any result changed to test the associations by omitting stages 1 and 2; nevertheless, no significant results were found either including or omitting stages 1 and 2. The chi-square and ANOVA tests were performed to measure the crude associations. Nevertheless, descriptive analysis showed that the high activity level had ‘small frequencies’ (1 participant); therefore, the data was combined with a moderate activity level, as the score of the participants was borderline to the high activity level category in multivariate analysis.

Meanwhile, in multivariate analysis, a general linear model (GLM) in the complex sampling was used to determine the association between the CTX and serum 25(OH)D concentrations, sex, place, ethnicity, dietary calcium intake, and physical activity, sun exposure, and sun-protective behavior adjusted for puberty. Linear regression analysis was performed for analyses with CTX as the dependent variable and 25(OH)D concentrations as independent variable. First, linearity was tested by categorizing the 25(OH)D concentration; if the regression coefficients of the categories of 25(OH)D did not decrease or increase linearly, the association was considered non-linear. Vitamin D status appeared non-linear, and therefore they were analyzed as categorical variables. Interaction was studied by adding the product term, 25(OH)D times calcium intake (mg/day), protein intake (energy %) or physical activity categories to the regression analyses. A *p* < 0.1 for the interaction term was considered significant.

Multicollinearity and model fit were assessed. The parathyroid (PTH) factor was omitted from the analysis as it is associated with serum 25(OH)D and calcium intake. The values presented the adjusted *β* values (estimated unstandardized regression coefficient) and the standard errors. The statistical tests and corresponding *p*-values were two-sided, and a p-value less than 0.05 was considered statistically significant.

## 3 Results

### 3.1 Participant characteristics

Initially, 1,234 participants enrolled in the present study. After excluding incomplete data and missing values, the total number of participants eligible for analysis was 1,136. The mean age of participants in this study was 15.1 ± 0.4 years old (range from 14.6 to 16.6 years old). No participant had a current endocrine disorder (e.g., hypoparathyroidism, hyperparathyroidism, rickets, osteoporosis, diabetes mellitus), took medications that affected bone metabolism, or had a systemic medical illness (e.g., chronic kidney disease, end-stage liver disease, malabsorption disease, malignancy). Table 1 tabulated the descriptive analyses of the participant characteristics in this study.

**Table 1:** Distribution of characteristics among 15-year-old participants by sex.

Characteristics by	Prevalence, n (%)	Male, n (%)	Female, n (%)
Location			
Urban	661 (58.2)	234 (53.3)	427 (61.3)
Rural	475 (41.8)	205 (46.7)	270 (38.7)
Ethnicity			
Malays	929 (81.8)	361 (82.4)	568 (81.4)
Non-Malays	207 (18.2)	77 (17.6)	130 (18.6)
Physical activity			
Low	640 (56.3)	185 (42.1)	455 (65.3)
Moderate & High	496 (43.7)	254 (57.9)	242 (34.7)
Body fat percentage			
Non-obese	873 (76.8)	358 (80.8)	515 (74.3)
Obese	263 (23.2)	85 (19.2)	178 (25.7)
CTX log concentrations (ng/mL) (mean±SD)	1.53 ± 0.75	1.24 ± 0.44	1.32 ± 0.47
Calcium Intake (mg/day)			
≤ 347.3 mg/day	325 (28.6)	95 (24.5)	230 (35.9)
347.4–412.5 mg/day	544 (47.9)	216 (55.8)	328 (1.2)
≥ 412.6 mg/day	159 (14.0)	76 (19.6)	83 (12.9)
25-hydroxyvitamin D (25(OH)D) concentration			
≤37.4 nmol/L	144 (12.7)	9 (22.0)	135 (20.6)
37.5–49.9 nmol/L	580 (51.1)	113 (23.4)	467 (71.3)
≥ 50 nmol/L	412 (36.2)	359 (74.6)	53 (8.1)
Parathyroid concentration (pmol/L) (mean±SD)	5.16 ± 3.35	5.79 ± 3.52	4.77 ± 3.18
Pubertal status			
Stage 1	5 (0.5)	2 (0.5)	3 (0.5)
Stage 2	10 (0.9)	3 (0.8)	7 (1.1)
Stage 3–4	873 (82.1)	326 (81.7)	531 (81.9)
Stage 5	175 (16.5)	68 (17.0)	107 (16.5)
Timing of outdoor activities			
7 a.m.–10 a.m.	160 (14.1)	55 (17)	105 (18)
10 a.m.–2 pm	55 (4.8)	19 (5.9)	36 (6.2)
2 pm–4 pm	198 (17.4)	54 (16.7)	144 (24.7)
4 pm–7 p.m.	494 (43.5)	196 (60.5)	298 (51.1)
Skin exposure			
Hands and face	440 (38.7)	51 (12.6)	389 (58)
Short sleeves	452 (39.8)	243 (59.9)	209 (31.1)
Shorts and t-shirt or short only	185 (16.3)	112 (27.6)	73 (10.9)
How often do you wear sunscreen?			
Never	702 (61.8)	324 (79.8)	378 (56.3)
Sometimes	325 (28.6)	78 (19.2)	247 (36.8)
Always	50 (4.4)	4 (1)	46 (6.9)
How often do you wear a shirt with long sleeves that cover your shoulders?			
Never	101 (8.9)	59 (14.5)	42 (6.2)
Sometimes	630 (55.5)	319 (78.2)	311 (46.2)
Always	350 (30.8)	30 (7.4)	320 (47.5)
How often do you wear a hat/tudung?			
Never	642 (56.5)	161 (40)	481 (72.1)
Sometimes	391 (34.4)	217 (53.8)	174 (26.1)
Always	37 (3.3)	25 (6.2)	12 (1.8)
How often do you stay in the shade or under an umbrella?			
Never	152 (13.4)	61 (14.9)	91 (13.7)
Sometimes	725 (63.8)	277 (67.7)	448 (67.3)
Always	198 (17.4)	71 (17.4)	127 (19.1)
How often do you wear sunglasses?			
Never	670 (59.0)	220 (54.1)	450 (67.8)
Sometimes	362 (31.9)	168 (41.3)	194 (29.2)
Always	39 (3.4)	19 (4.7)	20 (3)

Most of the participants were female adolescents (62%, *n* = 697), Malays (82%, *n* = 929), and 23% (*n* = 259) of the participants were obese. The mean CTX concentrations of the participants in this study were 1.53 ± 0.75 pg./mL. Females had a higher mean CTX concentration compared to males (mean ± SD; 2.32 ± 0.47 vs. 2.24 ± 0.44). Regarding the calcium intake of the participants, most of the participants were below 50% of the recommended nutrient intake, with a minimum of 282.0 mg/day and a maximum of 543.1 mg/day (Recommended Nutrient intake for dietary calcium; 1,000 mg/day; Table 1).

The study revealed that the prevalence of vitamin D deficiency (25(OH)D < 50 nmol/L) was 63.8%, with females having a higher rate than males (91.9% vs. 45.4%). About 70% (*n* = 692) of the participants spent time outdoors after 2 p.m. (e.g., after school; Table 1). Only 5% (*n* = 55) of participants spent their time outdoors from 10 a.m. to 2 p.m., the period with the most significant potential for vitamin D synthesis. Thirty-nine per cent (*n* = 440) of the participants wore concealing clothes that only exposed their hands and face (less skin exposure). This group (less skin exposure) had a slightly higher proportion of lower vitamin D concentration than those with more skin exposure, such as wearing short sleeves or shorts (59.1%, *n* = 205 vs. 40.9%, *n* = 142). Meanwhile, Table 2 shows the detailed distribution of CTX concentrations by participants’ characteristics.

**Table 2:** Mean of CTX (log concentrations) by sample characteristics.

Variables	Frequency (n)	Mean ± SD/ (ng/mL)
Sex		
Male	439	1.24 ± 0.44
Female	697	1.32 ± 0.47
Location		
Urban	661	2.32 ± 0.46
Rural	475	2.24 ± 0.46
Ethnicity		
Malays	929	2.26 ± 0.45
Non-Malays	205	2.41 ± 0.47
Physical activity score		
low	640	2.29 ± 0.47
moderate & high	496	2.28 ± 0.45
Body fat percentage		
non-obese	873	2.29 ± 0.46
Obese	263	2.27 ± 0.45
Calcium Intake		
≤ 347.3 mg/day	325	2.31 ± 0.49
347.4–412.5 mg/day	544	2.30 ± 0.45
≥ 412.6 mg/day	159	2.22 ± 0.48
25-hydroxyvitamin D (25(OH)D) concentration		
≤ 37.4 nmol/L	144	2.26 ± 0.48
37.5–49.9 nmol/L	218	2.28 ± 0.44
≥ 50 nmol/L	758	2.29 ± 0.47
Pubertal status/Tanner staging		
Stage 1	5	2.12 ± 0.31
Stage 2	10	2.19 ± 0.41
Stage 3–4	873	2.26 ± 0.45
Stage 5	175	2.29 ± 0.46
Timing of outdoor activities		
7 a.m.–10 a.m.	160	2.24 ± 0.45
10 a.m.–2 pm	55	2.31 ± 0.40
2 pm–4 pm	198	2.35 ± 0.47
4 pm–7 p.m.	494	2.27 ± 0.47
Skin exposure		
Hands and face	440	2.30 ± 0.47
Short sleeves	452	2.28 ± 0.45
Shorts and a t-shirt or shorts only	185	2.30 ± 0.48
How often do you wear sunscreen?		
Never	702	2.29 ± 0.45
Sometimes	325	2.29 ± 0.49
Always	50	2.35 ± 0.47
How often do you wear a shirt with long sleeves that cover your shoulders?		
Never	101	2.26 ± 0.39
Sometimes	630	2.29 ± 0.46
Always	350	2.30 ± 0.48
How often do you wear a hat/tudung?		
Never	642	2.30 ± 0.46
Sometimes	391	2.27 ± 0.46
Always	37	2.35 ± 0.50
How often do you stay in the shade or under an umbrella?		
Never	152	2.30 ± 0.46
Sometimes	725	2.29 ± 0.46
Always	198	2.29 ± 0.49
How often do you wear sunglasses?		
Never	670	2.31 ± 0.46
Sometimes	362	2.26 ± 0.47
Always	39	2.34 ± 0.43

### 3.2 Association between bone turnover marker [C-terminal telopeptide (CTX)], calcium intake and 25(OH)D concentrations in adolescents

The multivariate analysis revealed a higher bone turnover C-terminal telopeptide (CTX) concentrations with low calcium intake (≤ 347.3 mg/day; *β* = 0.161, *p*-value < 0.01 and 347.4–412.5 mg/day; *β* = 0.149, *p*-value < 0.01) and no association was observed between serum CTX and 25(OH)D concentrations (Table 3). However, the analysis showed that adolescents living in urban residents (*β* = 0.176, *p*-value < 0.01), avoiding sun-protective behavior (never / sometimes applied sunscreen; *β* = −0.280, *p*-value < 0.01 / *β* = − 0.248, *p*-value < 0.01; never wore sunglasses *β* = 0.187, *p*-value < 0.01; never wore hijab/hat *β* = − 0.212, *p*-value < 0.05), and participating in outdoor activities from 2 p.m. to 4 p.m. (*β* = 0.112, *p*-value < 0.05) were significantly associated with the serum CTX concentrations (Table 3).

**Table 3:** Association of participants characteristics, (25OH)D concentrations and calcium intake toward the bone turnover marker [C-terminal telopeptide (CTX)] in adolescents.

Characteristics by	Model 1			Model 2			Model 3			Model 4			Model 5			Model 6		
	*β*	95% CI		*β*	95% CI		*β*	95% CI		*β*	95% CI		*β*	95% CI		*β*	95% CI	
		LB	UB		LB	UB		LB	UB		LB	UB		LB	UB		LB	UB
Gender																		
Male	−0.076**	−0.14	−0.09	−0.048	−0.12	0.024	−0.061	−0.135	−0.014	−0.066*	−0.143	0.011	−0.060	−0.145	0.026	0.000	−0.093	0.092
Female (ref)																		
Place																		
Urban	0.145***	0.09	0.21	0.137***	0.074	0.201	0.16***	0.093	0.224	0.169***	0.096	0.241	0.169***	0.096	0.242	0.176***	0.102	0.250
Rural (ref)																		
Ethnicity																		
Malays	−0.038	−0.04	0.114	−0.019	0.062	0.101	−0.009	−0.077	0.096	0.009	−0.081	0.100	0.041	−0.056	0.138	0.065	−0.035	0.165
Non-Malays (ref)																		
25-hydroxyvitamin D level																		
Less than 37.4	−0.043	−0.15	0.06	−0.03	−0.14	0.08	−0.036	−0.148	0.077	−0.036	−0.151	0.079	−0.034	−0.149	0.081	−0.037	−0.153	0.079
37.5–49.9	−0.004	−0.07	0.08	0.014	−0.07	0.10	−0.011	−0.096	0.075	−0.023	−0.115	0.069	−0.026	−0.120	0.068	−0.033	−0.122	0.056
More than and equal to 50.0 (ref)																		
Body fat (obesity)																		
Non-obese				0.014	−0.07	0.09	−0.001	−0.086	0.085	−0.001	−0.094	0.091	−0.004	−0.098	0.090	−0.028	−0.123	0.067
Obese (ref)																		
Physical activity																		
Low				0.020	−0.05	0.09	0.024	−0.047	0.094	0.041	−0.035	0.117	0.041	−0.037	0.118	0.060	−0.016	0.135
Moderate & high (ref)																		
Calcium Intake																		
≤ 347.3 mg/day				0.09*	−0.011	0.19	0.092*	−0.013	0.197	0.135**	0.027	0.243	0.143**	0.033	0.252	0.161***	0.052	0.270
347.4–412.5 mg/day				0.08*	−0.010	0.18	0.084*	−0.011	0.180	0.119**	0.022	0.215	0.125**	0.039	0.226	0.149***	0.054	0.243
≥ 412.6 mg/day (ref)																		
Tanners Staging																		
Stage 1 & 2							−0.050	−0.22	0.12	−0.041	−0.195	0.114	−0.047	−0.304	0.003	−0.039	−0.213	0.136
Level 3 & 4							0.024	−0.073	0.12	0.052	−0.053	0.158	0.048	−0.103	0.089	0.043	−0.067	0.153
Level 5 (ref)																		
Timing outdoor																		
7 a.m.-10 a.m.										0.034	−0.078	0.146	0.033	−0.082	0.098	0.022	−0.081	0.126
10 a.m.-2 pm										−0.029	−0.161	0.104	−0.033	−0.102	0.179	−0.051	−0.185	0.083
2 pm-4 pm										0.132***	0.043	0.222	0.129***	0.013	0.178	0.112**	0.022	0.203
4 pm 7 p.m. (ref)																		
Skin Exposure																		
Hands and face													0.047	−0.066	0.167	0.031	−0.092	0.155
Short sleeves													0.068	−0.044	0.179	0.062	−0.049	0.172
Shorts and t-shirt or shorts only (ref)																		
How often do you wear sunscreen																		
Never																−0.280***	−0.454	−0.107
Sometimes																−0.248***	−0.426	−0.070
Always (ref)																		
How often do you wear a shirt with long sleeves that cover your shoulders																		
Never																−0.125	−0.288	0.037
Sometimes																−0.050	−0.146	0.047
Always (ref)																		
How often do you wear a hat/tudung																		
Never																−0.212**	−0.405	−0.020
Sometimes																−0.200**	−0.398	−0.002
Always (ref)																		
How often do you stay in the shade or under an umbrella																		
Never																0.056	−0.077	0.189
Sometimes																−0.015	−0.122	0.092
Always (ref)																		
How often do you wear sunglasses																		
Never																0.187***	0.046	0.327
Sometimes																0.155**	0.014	0.296
Always (ref)																		

*, **, *** Significant at 10, 5 and 1%; LB, lower bound; UB, upper bound; CI, Confidence interval.

## 4 Discussion

This study examined the associations between dietary calcium intake, serum 25(OH)D concentrations, and bone turnover (CTX) among Malaysian adolescents. Current findings showed that low calcium intake was significantly associated with higher CTX levels, whereas vitamin D deficiency, although highly prevalent, was not associated with CTX. This result is consistent with the biological role of calcium as the main substrate for bone mineralization, where inadequate intake may directly increase bone resorption. In contrast, the lack of association between 25(OH)D and CTX may reflect that vitamin D concentrations were uniformly low in this population, possibly below the threshold at which variability in bone turnover could be detected. Previous studies in European and Asian cohorts (11, 15, 26) reported similar findings, emphasizing the dominant role of calcium intake in regulating bone turnover during adolescence. However, some studies acknowledged that lacking vitamin D leads to defective bone formation and mineralization, including increasing bone resorption and ultimately increasing the CTX concentrations (4, 27, 28). While, Suriawati et al. (18) conducted a study to investigate the relationship between the dietary intake, vitamin D and bone mineral content (BMC) in 13-year-old Malaysian adolescents and reported that those subjects with a higher intake of vitamin D and a higher combination of the intake of vitamin D and calcium resulted in significantly higher BMC quartiles. Current study, where the outcome was the CTX concentrations, found that higher bone turnover CTX concentrations were not significantly associated with low serum 25(OH)D among adolescents with low calcium intake. The potential explanation involves the optimal PTH concentration in serum that may be associated with vitamin D concentrations, whereby in this study, the average PTH concentration among adolescents was only 5.16 ± 3.35 pmol/L, which is borderline the normal range concentration.

Though the serum CTX concentration is a sensitive biomarker of bone resorption because it responds quickly to bone metabolism changes (26), and vitamin D is essential for bone formation and mineralization (4, 5), a study by Marwaha et al. (11) suggested that bone remodeling requires optimal concentrations of serum PTH. A low concentration of PTH may be associated with low bone formation and high concentrations of PTH with high bone turnover. Hence, suppression of PTH with vitamin D supplementation is associated with decreased bone resorption, as observed in their vitamin D supplementation study (11). On the other hand, a few studies suggested that the 25(OH)D is associated with bone turnover only when the calcium intake is above the median intake. In contrast, not when the calcium intake is lower than the median (29–31). The present study population’s calcium intake was also low, less than 50% of the recommended nutrient intake. Together with the prevalence of vitamin D deficiency (25(OH)D < 50 nmol/L) was high, 63.8% with females had a higher rate than males (91.9% vs. 45.4%), and the concentration of PTH of the study population also borderline average range concentration, thus, these conditions could be possible explanations for the absence of impact between 25(OH)D and CTX concentrations in the present study.

Nevertheless, apart from low calcium intake, the multivariate analysis revealed that the factor of urban residents having outdoor activities between 2 p.m. and 4 p.m., sunscreen avoidance, and always wearing a hijab/hat were significantly associated with a higher concentration of CTX among adolescents in this study. The current results showed that adolescents who live in urban areas had been found to have higher CTX concentrations than those living in rural areas. It is known that urbanization has changed dietary patterns and lifestyles, leading urban adolescents to a sedentary lifestyle and being less physically active, lacking exercise, improper nutrition, and tobacco and alcohol use (32). These health pattern changes have been linked to the risks of many diseases, including impacting bone turnover markers, indicating increased concentrations of bone resorption. Furthermore, low calcium intake and 25(OH)D concentrations among adolescents in this study may not suffice to provide additional calcium to control the homeostasis process (28).

Our results also found that those who had outdoor activities between 2 p.m. and 4 p.m. had higher CTX concentrations. This contrasted with previous studies (33, 34). Theoretically, exposure to sunlight between 10.00 a.m. and 4.00 p.m. would absorb ultraviolet light to ensure that vitamin D is sufficient to maintain bone health functions and calcium balance. It is essential to bone formation and mineralization (16, 17). It has been suggested that approximately 20–30 min of sun exposure between 10.00 a.m. and 4.00 p.m. at least twice a week to the face, arms, legs, or back without sunscreen will usually provide sufficient daily vitamin D requirements (33, 35). Thus, reducing bone resorption and lowering bone resorption markers (e.g., CTX).

Nevertheless, sun-protection behavior and clothing styles (such as long sleeves, long pants, and wearing a hijab/hat) could eliminate ultraviolet absorption and lower vitamin D concentrations. This was in line with the present findings. The present study found that those who had sunscreen avoidance behaviors and never wore hijab/hat had lower CTX concentrations than those who applied for sunscreen protection. These explanations could be why our study found that those who had outdoor activities between 2 p.m. and 4 p.m. had higher CTX concentrations. Additionally, as most of the participants in this study were females and Malays, these factors (sunscreen avoidance and clothing styles) were synonyms. Malaysian women, like some Asian populations, prefer to have fairer skin (34). Therefore, although they had outdoor activities between 10.00 a.m. and 4.00 p.m., they might apply sunscreen protective behavior, concealing their skin with long sleeves and long pants and wearing a hat.

The present study found that the calcium intake among adolescents was lower than the recommended Malaysian adolescent nutrient intake, which is in line with previous studies that reported low calcium intake among Malaysian adolescents due to a low intake of milk (18). A few studies have shown milk consumption was positively correlated with decreasing the serum CTX concentration in adolescents (36–39). This is comparable to the present results that a low calcium intake is associated with higher CTX concentrations. The relatively low calcium intake observed in this study may account for the lack of significant association between higher vitamin D levels and increased CTX concentration (*p* = 0.127). This could be attributed to the dual role of vitamin D. When there is an adequate supply of calcium, vitamin D and its metabolites can enhance the calcium balance and promote mineral deposition in the bone matrix. Conversely, in the presence of calcium deficiency, 1,25(OH)2D may stimulate bone resorption while concurrently inhibiting bone mineralization to maintain serum calcium homeostasis, potentially at the expense of bone mass (31, 40). This emphasizes the importance of a sufficient calcium intake. In addition, in the Malaysia National Health and Morbidity Survey 2022, only 23.2% of adolescents consumed milk / milk products at least two times daily (41).

This is the first study investigating the association of bone turnover (CTX) with low calcium intake and 25(OH)D concentrations among adolescents in Malaysia. In addition, to our knowledge, this study had a larger sample of Southeast Asia investigating the CTX bone turnover marker among adolescents. Current study supports inadequacy of both calcium and vitamin D insufficiency are highly prevalent. This large, multi-ethnic adolescent sample from middle income country will complement the existing literature from Western and Northern Asian populations. The inclusion of PTH as a covariate in our analysis further strengthens the robustness of our findings. PTH is a central regulator of calcium and vitamin D metabolism, and its inclusion confirmed that inadequate calcium intake remained significantly associated with higher CTX levels independent of PTH concentrations. These data are essential for future intervention studies which aim to decrease bone resorption. However, several limitations should be considered in the interpretation of the present study. First, the cross-sectional design precludes determinations of cause and effect. Second, dietary intake was based on self-reported 7-day diet history interviews, which may be subject to recall bias (20, 42). However, trained dietitians conducted interviews where audit checks were in place, and the method has been validated in Malaysian adolescents. However, vitamin D supplementation data were not collected, although this is unlikely to have significantly affected results for this age group as current Malaysia Ministry of Health policy is focusing on food first dietary-based approach. Despite these limitations, our study has important public health implications. The finding that calcium intake, rather than vitamin D deficiency, is associated with elevated bone turnover highlights the urgent need for interventions to increase calcium intake among Malaysian adolescents. Strategies such as school-based nutrition programs, expansion of food fortification program, and ensuring the affordability of calcium-rich foods may help to improve bone health outcomes in this vulnerable age group. Lastly, it should be noted that the recruitment of students involved only those attending national schools because more than 85% of Malaysian students go to national schools. Therefore, these findings cannot be generalized to all Malaysian adolescents as it does not include participants from east Malaysia.

## 5 Conclusion

In conclusion, this study found that low dietary calcium intake, rather than vitamin D deficiency, was significantly associated with elevated CTX levels, a marker of bone resorption, among Malaysian adolescents. This is one of the first large-scale studies in Southeast Asia to explore these associations, providing empirical evidence from a multi-ethnic Malaysian adolescent cohort. This study also highlights the urgent need for public health strategies that promote calcium intake through improved dietary habits, food fortification initiatives, and affordable access to calcium-rich foods. Longitudinal and interventional studies are warranted to confirm these associations and to explore the combined role of calcium and vitamin D in optimizing adolescent bone health. Further research is warranted to ascertain the determinants of CTX concentrations.

## Data Availability

Data supporting the findings of this study are available upon reasonable request from the corresponding author MHA due to ethical reason as participants are children.
